# Enhanced structural integrity of Laser Powder Bed Fusion based AlSi10Mg parts by attaining defect free melt pool formations

**DOI:** 10.1038/s41598-023-43718-2

**Published:** 2023-10-04

**Authors:** M. Saravana Kumar, Che-Hua Yang, Muhammad Umar Farooq, V. Kavimani, Adediran Adeolu Adesoji

**Affiliations:** 1 Graduate Institute of Manufacturing Technology, National Taipei University of Technology, Taipei, 10608 Taiwan; 2 School of Mechanical Engineering, University of Leeds, Leeds, LS2 9JT UK; 3 Department of Mechanical Engineering, Karpagam Academy of Higher Education, Coimbatore 21, Tamil Nadu India; 4 Department of Mechanical Engineering, Landmark University, P.M.B. 1001, Omu-Aran, Kwara State Nigeria; 5 MTE Division, CSIR-National Metallurgical Laboratory, Jamshedpur, India; 6 Department of Mechanical Engineering Science, University of Johannesburg, Auckland Park Kingsway, Johannesburg, South Africa

**Keywords:** Materials for devices, Mechanical engineering

## Abstract

This research aims to fabricate an AlSi10Mg parts using Laser Powder Bed Fusion technique with enhanced structural integrity. The prime novelty of this research work is eliminating the balling and sparring effects, keyhole and cavity formation by attaining effective melt pool formation. Modelling of the Laser Powder Bed Fusion process parameters such as Laser power, scanning speed, layer thickness and hatch spacing is carried out through Complex Proportional Assessment technique to optimize the parts' surface attributes and to overcome the defects based on the output responses such as surface roughness on scanning and building side, hardness and porosity. The laser power of 350 W, layer thickness of 30 µm, scan speed of 1133 mm/s, and hatch spacing of 0.1 mm produces significantly desirable results to achieve maximum hardness and minimum surface roughness and holding the porosity of < 1%. The obtained optimal setting from this research improves the structural integrity of the printed AlSi10Mg parts.

## Introduction

The growing demand for metal parts with complicated shapes and excellent quality has fueled the advancement of innovative manufacturing technology. Laser Powder Bed Fusion (L-PBF), one of the most common metal additive manufacturing processes, is well renowned for its capacity to produce high-quality, defect-free complicated parts^1^. Till now, a wide range of alloys were fabricated by LPBF process, namely, steels, scalmalloy, titanium alloys and aluminum alloys^2^. Moreover there alloys shows similar corrosion resistance when compared to their conventionally manufactured counter parts^3^. Al-Si alloys have various advantages, including light weight, strong wear resistance, good thermal expansion coefficient, high specific strength, and good thermal conductivity, making them widely employed in the automobile and aerospace industries^4^. Mechanical properties are well known to be sensitive to microstructure, including phase components, grain size and morphologies, dendritic and elemental segregation^5^. As a result, microstructure manipulation and optimization is a well-known approach for achieving appropriate mechanical characteristics for industrial purposes. Naoki Takata et al. compared the heat treated AlSi10Mg parts with the as build parts based on the microstructural orientations. They have found that the several columnar grains of Al matrix was surrounded by the fine Si-phases and after the heat treatment, there occurs the transformation of columnar grains to equiaxed grains by showing [001] texture. These observation reflects in the mechanical properties by showing the maximum tensile strength of 480 MPa^6^. Qian et al. compared the mechanical properties of the AlSi10Mg parts which was fabricated by LPBF and casting process. The enhanced mechanical properties were observed in the LPBF based AlSi10Mg parts which was due to the occurrence of precipitate formation during rapid heating and cooling process in the LPBF technique. However, there was an lower elongation of LPBF based AlSI10Mg parts when compared with the casted parts which was due to the strong melt pool grain boundary characteristics^7^. Liu et al. used the machine learning technique in the LPBF process to examine the microstructure and fracture mode based on the process parameters. However, they have focused to achieve both strength and ductility by optimizing the parameters. They have suggested that the optimal setting of laser power and scanning speed helps to achieve maximum relative density of more than 99%^8^. Silvestri et al. focused to analyze the range in mechanical properties of AlSi10Mg parts based on the various manufacturing condition of different machines. Even though, some researchers were trying to solve this problem by increasing the repeatability, different manufacturer and SLM machines were also plays some role. They have found out the range of tensile strength of the AlSi10Mg parts by performing the printing process on the various SLM machines^9^. Erfan Maleki et al. stressed that different post-processing operations, such as shot peening, ultrasonic nanocrystalline surface modification, severe vibratory peening, electrochemical polishing, and other hybrid treatments obtained from their combination were studied on the microstructure, surface, and mechanical properties of LPBF AlSi10Mg specimens to correct various surface flaws. They also came to the conclusion that the hybrid mechanical and chemically treated specimens were significantly more wettable and had significantly lower surface roughness after electro-chemical polishing^10^. The intermittent electrochemical polishing (IECP) method, according to Han Liu et al., improved the polishing quality of LPBF AlSi10Mg. They discovered a novel mechanism for the formation of viscous layers on the LPBF AlSi10Mg surface during the Electro Chemical Polishing process^11^. Patakham et al. emphasize the role of Si phase and melt pool formation on the elastic and fracture behavior of the AlSi10Mg parts and also examined the effect of print orientation and heat treatment on the tensile strength of the printed parts. The tensile test results confirms the anisotropic property of the printed parts by showing different tensile strength in different directions^12^. Liu et al. aimed to produce gradient in the microstructure to improve the mechanical and wear strength of the printed parts which was mainly attributed by the cooling rate during the melt pool formation. They have noted that cooling rate in the surface was much higher when compared with the cooling rate at the bottom. This gradation in the cooling rate not only improves the morphology but also contributes more towards the mechanical properties^13^.

Few researchers adopted Finite Element Simulation techniques to validate the mechanical properties of the printed parts. Anne Mertens et al. investigated the thermal history of laser powder bed fusion based AlSi10Mg parts by examine their microstructure and mechanical properties. They have analyzed the interaction of solute Si with respect to volume and size in the Al matrix and used the Finite Element Simulation to validate the theoretical findings based on the Rosenthal’s and Matyja’s equations^14^. Shiwen Liu et al. focused on the microstructural formation in the single track based on the thermal variables and the transformation of columnar to equiaxed transition. Similarly, finite element analysis was adopted to interpret the thermal variables. Finally, they have concluded by comparing the results of melt pool characteristics from the experimental data and simulated data^15^. Chen et al. adopted the Finite Element Simulation technique to examine the porosity and mechanical properties of the LPBF based AlSi10Mg parts. They have analyzed the melt pool formation and thermal history on the perspective to produce an optimum energy density and also confirms the anisotrophy of the printed parts by inspecting the growth characteristics of the Si phases. The results confirms that the optimized parameters aided in reducing then porosity of the printed AlSi10Mg parts^16^. Xihe Liu et al. explore the texture component in AlSi10Mg parts through EBSD analysis. They have noted a pseudoeutectic structure with fibrous Si network and suggested that was due to the higher cooling rate and also three different orientation of microstructure in the melt pool region was illustrated by the EBSD analysis. The results confirms the formation of coarse grains during the re-melting stage^17^. Wang et al. examined the pores formation in the LPBF based AlSi10Mg parts and also examine their mechanism behind their pores formation. They have found that different types of pores were located such as irregular pores and circular pores and these pores were caused by the entrapment of gases or may be due to the vaporization during the heating and cooling process in the LPBF technique^18^. Yang Liu et al. used Taguchi design of experiments to examine the impact of laser power, scanning speed, powder feeding rate and shielding gar flow on the relative density of the AlSi10Mg printed parts by powder delivered Laser Deposition Process (LDP). They can able to build AlSi10Mg part with more than 99% density with the help of optimized parameters^19^.

From the literature survey, it was verified that, most of the research was focused on the microstructural gradient and mechanical characteristics of AlSi10Mg parts. But, there was a lack of studies on surface characteristics of the LPBFed AlSi10Mg parts and that would be a beneficial studies in the perspective of industrial applications which helps to build the defect free parts with improved dimensional accuracy. Hence, this research focus to improve the surface qualities by enhancing the surface roughness and hardness by reducing porosity, balling effect, spattering effect and key hole formation. Four major parameters (Laser power, scanning speed, layer thickness and hatch spacing) were considered in this study and these parameters were examine based on the four responses (SS surface roughness and BS surface roughness, hardness and porosity percentage) with the help of COPRAS multi-objective optimization technique. This outcome of this research mainly focus on removing the balling and spattering effects, keyhole formation, reducing porosity and also on attaining effective melt pool formation.

## Materials and methods

### Powder properties

**Figure 1:**
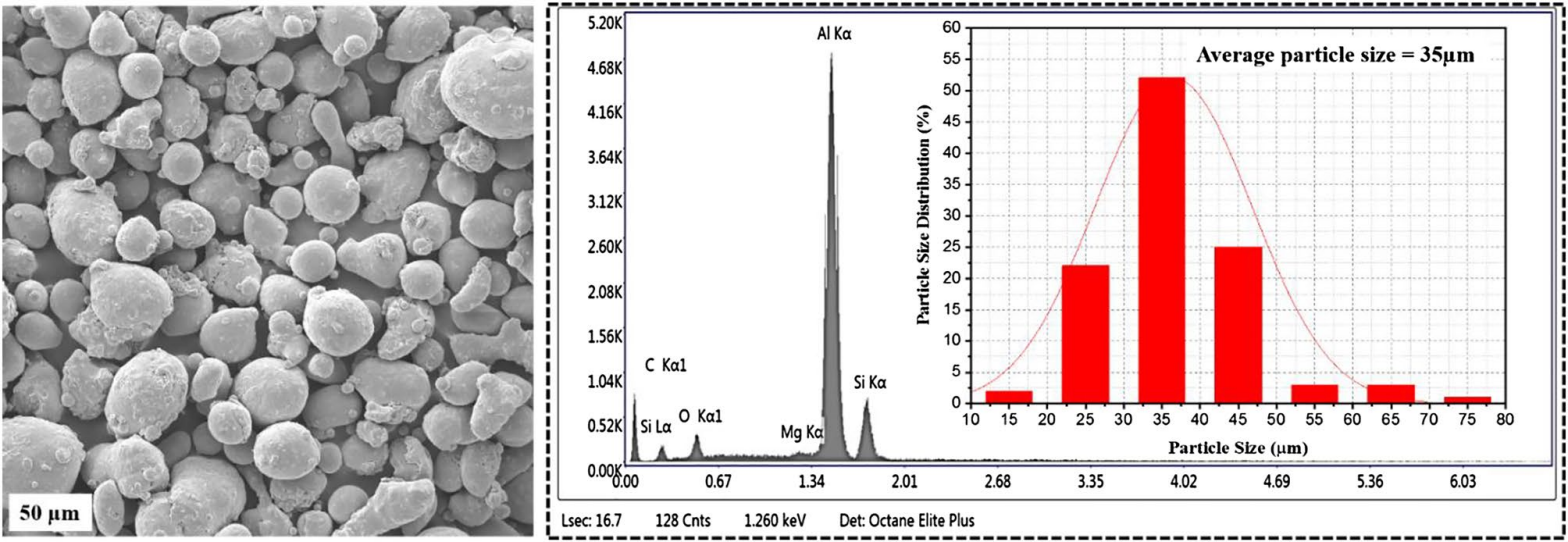
Particle size distribution plots with EDX results of the AlSi10Mg powder.

The LPBF printing process was mainly influenced by the properties of the powder. The AlSi10Mg powder used in the process has the size range of 25–65 μm and that was confirmed in the particle size distribution plot in Fig. 1. The SEM analysis of the particle size confirms that the particles are spherical in shape. This spherical shape of the AlSi10Mg powder improves the flow ability^20^. The EDX analysis from the powder also approves the occurrence of major elements such as Al (90–91%), Mg (0.2–0.4%) and Si (9–11%), this standard range of major elements confirms the purity of the AlSi10Mg powder. More over the presence of more amount of Mg elements enhances the solid solution strengthening effect as well as it increases the hardness of the printed AlSi10Mg parts^21^. The sudden rise of oxygen content in the printing chamber may occur due to the gas entrapment in the AlSi10Mg powder particles^22^. This results in the fluctuation in the density of the printed parts^23^. So, the power particles were ensured for the occurrence of gas entrapments before feeding in the printing chamber. Further, the EDS analysis displays the elemental composition of AlSi10Mg powder in Table 1.

**Table 1:** Elemental composition of AlSi10Mg powder.

Elements	Al	Si	Mg	Fe	Mn	Ni	Cu	Ti
Weight %	90.46	9	0.32	0.13	0.04	0.013	0.017	0.013

### LPBF and the parameters

**Figure 2:**
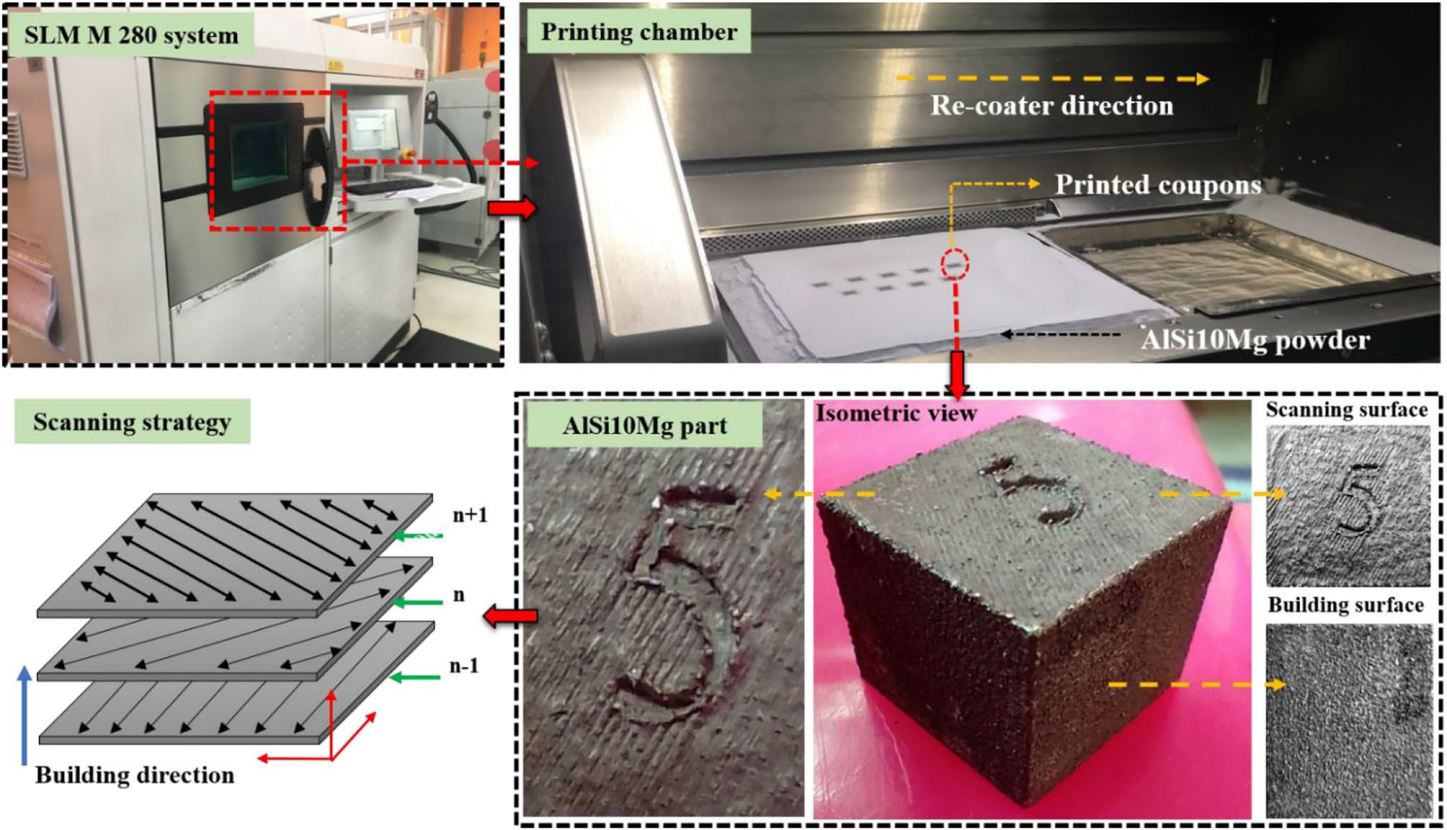
Schematic representation of L-PBF process with the printed AlSi10Mg parts.

AlSi10Mg samples were fabricated using the LPBF technique in the EOS M280 system which has 7,000 hPa of compressed air supply; 20 m^3^/h and Ytterbium fibre laser with a nominal power of 400W was used to melt the powder and the laser beam has a diameter of 100 to 500 μm at the building area. To lessen thermal stress and sample deformation, the platform was heated to 150 °C before melting. To stop the oxidation of the aluminum powder, the material was melted in an argon-filled forming chamber with a decreased oxygen level < 0.01%. To distinguish samples of various parameters, labels of 0.5 mm depth were printed in the upper right corner of each sample. The production of cubic samples (10 × 10 × 10 mm^3^) involved a 67° rotational scanning mode. After the samples were printed, Wire Electric Discharge Machining (WEDM) isolated the samples from the platform. Figure 2 shows the EOS M280 system and the printing of AlSi10Mg parts.

### Experimental design

For this investigation, the experiments were designed using Box Behnken Design (BBD) with three different levels for four input factors: Laser power, scanning speed, layer thickness and hatch spacing which was shown in Table 2 and also four output parameters: scanning side (SS) surface roughness and building side (BS) surface roughness, surface hardness and porosity percentage was considered to examine the influence of printing process parameters on the surface characteristics of the printed AlSi10Mg parts.

**Table 2:** Investigated and fixed parameters.

Selected Parameters	Levels			Fixed parameters	
	Low	Middle	High		
Layer power (W)	250	300	350	Print orientation	Vertical
Hatch spacing (mm)	0.1	0.2	0.3	Scan strategy	(0.67)
Scan speed (mm/s)	1000	1300	1600	Build plate temperature (°C)	170
Layer thickness (µm)	30	40	50		

### Response measurement

The relative density of the printed parts can be used to estimate the porosity percentage (P% + RD = 100). Calculating relative density involves taking the bulk density and dividing it by the specimen's theoretical density. The bulk density of the printed parts were measured using the Archimedes methods^24^. Similarly, the rule of mixtures can be used to compute the theoretical density. Using Eq. (1), the sample' porosity was calculated.

1 $${\mathrm{Porosity}} ({\%})=1-\frac{{\rho \exp}}{{\rho {\text{theo}}}} \times 100$$

Following polishing, the cross-sections were electrolytically etched for 5 s at a direct current voltage of 32 V using a 1:3 solution of perchloric acid and ethanol^25^. From the polished and etched surface, various defects were examined using SEM with EDAX analysis (JEOL JSM-6380LA) and the interactions of various combinations of process parameters were interpreted.

Hardness test was performed based on the ASTM: E384 standards. During the micro hardness test, a load of 1 kg was applied to the Vickers (DPH) diamond indenter, which breaches the printed surface during the course of a 10-s dwell time. The hardness test was performed on both the top (Scanning surface) and side surface (building surface) to investigate the fluctuation in the hardness values. However, only the scanning surface's hardness was considered to examine the interaction of combined process parameters on the hardness of the printed AlSi10Mg parts. Three trials have been taken for each place and the average value from those trials was considered to draw the plots for both the surfaces. A microscope was used to carefully measure the diagonal indentation effect, and the load and indentation size were used to calculate the Vickers hardness.

Using a Taylor-Hobson Surtronic 3 + surface roughness tester with a 0.8 mm sampling length, surface roughness of the printed parts were examined. Top surface roughness were evaluated by taking the average of surface roughness along and across the scanning direction. Similarly, side surface roughness was taken from the average value from three different locations along the build direction.

## Results and discussion

### Parametric optimization

#### Parametric influence analysis

The parametric influence analysis is based on the analysis of variance where the significance of individual parameters and their interactions are determined^26,27^. The significance of the parameters is categorized with respect to confidence interval of 90% and 95%. The parameters and their interactions having *p*-value less than 0.05 are classified as significant factors to control respective response under 95% confidence. Whereas the *p*-value less than 0.1 shows 90% confidence of the parameter for influencing the response variable. The brief representation of the analysis of variance of the process parameters is shown in Table 3. The non-linear quadratic model was chosen to evaluate parameters’ interactive effects. The linear appears to be significant (p-value 0.005) whereas other squared or two-way parametric interactions are also found to be influential with 90% or 95% confidence^28,29^. Therefore, the selection of non-linear quadratic model is merely supported by the fact of comprehensive parametric evaluation.

**Table 3:** Analysis of variance of process parameters.

Source	Hardness		Porosity		Ra-Top		Ra-Side	
	F-value	p-value	F-value	p-value	F-value	p-value	F-value	p-value
Model	4.80	0.005*	2.92	0.035*	12.66	0.000*	15.24	0.000*
Linear	11.81	0.000*	7.50	0.003*	36.95	0.000*	52.07	0.000*
Laser power	18.54	0.001*	18.20	0.001*	92.38	0.000*	178.43	0.000*
Layer thickness	15.10	0.002*	8.24	0.014*	44.28	0.000*	22.52	0.000*
Scan speed	0.10	0.758	0.31	0.586	7.12	0.020*	0.05	0.835
Hatch spacing	13.51	0.003*	3.27	0.095**	4.03	0.068**	7.29	0.019*
Square	1.34	0.310	1.04	0.427	6.72	0.004*	0.72	0.594
Laser power*Laser power	2.71	0.126	3.61	0.082**	1.24	0.287	0.83	0.381
Layer thickness*Layer thickness	3.44	0.088**	0.00	0.967	2.69	0.127	0.00	0.992
Scan speed*Scan speed	0.15	0.707	0.70	0.418	20.03	0.001*	0.88	0.366
Hatch spacing*Hatch spacing	0.03	0.864	0.41	0.536	14.03	0.003*	0.26	0.621
2-Way Interaction	2.44	0.089**	1.12	0.404	0.42	0.853	0.36	0.893
Laser power*Layer thickness	0.83	0.381	0.04	0.838	0.31	0.589	0.50	0.491
Laser power*Scan speed	10.72	0.007*	0.01	0.905	0.45	0.514	0.45	0.516
Laser power*Hatch spacing	0.53	0.481	0.58	0.460	0.02	0.894	0.20	0.662
Layer thickness*Scan speed	1.19	0.297	0.38	0.549	0.68	0.426	0.88	0.367
Layer thickness*Hatch spacing	0.53	0.481	3.53	0.085**	0.89	0.365	0.00	0.991
Scan speed*Hatch spacing	0.83	0.381	2.19	0.165	0.16	0.693	0.10	0.753

*95% confidence level.

**90% confidence level.

The predictive model for analysis of hardness is significant (*p*-value 0.005) having linear effects significant (*p*-value 0.000) under 95% confidence and two-way interactions (*p*-value 0.089) under 90% confidence interval. The hardness of the printed sample is significantly controlled by laser power (*p*-value 0.001), layer thickness (*p*-value 0.002) and hatch spacing (*p*-value 0.003). Similarly, the joint effect of laser power and scan speed (*p*-value 0.007) significantly influence the hardness under 95% confidence interval. The squared interaction of layer thickness (*p*-value 0.088) also controls the hardness significantly. On the other hand, the model for porosity is significant with *p*-value of 0.035 having influential linear effects with *p*-value of 0.003. The effect of parameters on porosity is analogous to hardness. The major influential parameters on the porosity include laser power (*p*-value 0.001) and layer thickness (*p*-value 0.014). However, hatch spacing (*p*-value 0.095) and the combined effect of the layer thickness with hatch spacing (*p*-value 0.085), and the squared term of laser power (*p*-value 0.082) are found to be influential at 90% confidence interval.

In the case of top roughness, the major influential parameters are laser power (*p*-value 0.000), layer thickness (*p*-value 0.000), scan speed (*p*-value 0.020), and hatch spacing (*p*-value 0.068). The squared interactions of the scan speed (*p*-value 0.001) and hatch spacing (*p*-value 0.003) significantly control the top roughness. However, no interaction of parameters was found to be significant. In the case of side roughness, the controlling parameters are laser power (*p*-value 0.000), layer thickness (*p*-value 0.000), and hatch spacing. Conclusively, all the parameters and their levels need careful selection to optimize the build quality. A similar underlying reasoning was established by Rashia Begum et al.^30^. The laser power and layer thickness are found to be the most influential parameters under 95% confidence and hatch spacing under 90% confidence on all response indicators.

#### Empirical modelling of process parameters

The other dimension of the current study is to formulate empirical models of the input variables to calculate the response characteristics which has not been comprehensively focused before^31^. The wide parametric ranges are used for modelling through response surface methodology based experimental data. The empirical relations are shown in Eq. (2) for hardness, Eq. (3) for porosity, Eq. (4) for top roughness, and Eq. (5) for side roughness. The non-linear quadratic model for hardness is developed with R^2^ = 84.86% and Adj. R^2^ = 67.19%. Similarly, the model for porosity is proposed R^2^ = 77.33% and Adj. R^2^ = 50.88%.

2 $$\begin{aligned} {\text{Hardness}}\;({\text{VHN}}) & = 205.2 - 0.326{\text{ Laser}}\;{\text{power}} - 1.087{\text{ Layer}}\;{\text{thickness}} - 0.0570{\text{ Scan}}\;{\text{speed}} \\ &\quad + 18.3{\text{ Hatch}}\;{\text{spacing}} + 0.000392{\text{ Laser}}\;{\text{power}}*{\text{Laser}}\;{\text{power}} \\ &\quad + 0.01104{\text{ Layer}}\;{\text{thickness}}*{\text{Layer}}\;{\text{thickness}} + 0.000003{\text{ Scan}}\;{\text{speed}}*{\text{Scan}}\;{\text{speed}} \\ &\quad + 10.4{\text{ Hatch}}\;{\text{spacing}}*{\text{Hatch}}\;{\text{spacing}} - 0.00125{\text{ Laser}}\;{\text{power}}*{\text{Layer}}\;{\text{thickness}} \\ &\quad + 0.000150{\text{ Laser}}\;{\text{power}}*{\text{Scan}}\;{\text{speed}} - 0.100{\text{ Laser}}\;{\text{power}}*{\text{Hatch}}\;{\text{spacing}} \\ &\quad + 0.000250{\text{ Layer}}\;{\text{thickness}}*{\text{Scan}}\;{\text{speed}} + 0.500{\text{ Layer}}\;{\text{thickness}}*{\text{Hatch}}\;{\text{spacing}} \\ &\quad - 0.0208{\text{ Scan}}\;{\text{speed}}*{\text{Hatch spacing}} \\ \end{aligned}$$

$${\text{R-sq}} = 84.86\% ,{\text{R-sq}}\left( {{\text{adj}}} \right) = 67.19\%$$

3 $$\begin{aligned} {\text{Porosity}} & = - 31.4 + 0.166{\text{ Laser}}\;{\text{power}} + 0.411{\text{ Layer}}\;{\text{thickness}} + 0.0104{\text{ Scan}}\;{\text{speed}} \\ &\quad - \, 1.3{\text{ Hatch}}\;{\text{spacing}} - 0.000379{\text{ Laser}}\;{\text{power}}*{\text{Laser power}} \\ &\quad - \, 0.00021{\text{ Layer}}\;{\text{thickness}}*{\text{Layer}}\;{\text{thickness}} - 0.000005{\text{ Scan}}\;{\text{speed}}*{\text{Scan}}\;{\text{speed}} \\ &\quad - \, 31.8{\text{ Hatch}}\;{\text{spacing}}*{\text{Hatch}}\;{\text{spacing}} + 0.00024{\text{ Laser}}\;{\text{power}}*{\text{Layer}}\;{\text{thickness}} \\ &\quad + \, 0.000005{\text{ Laser}}\;{\text{power}}*{\text{Scan}}\;{\text{speed}} + 0.088{\text{ Laser power}}*{\text{Hatch spacing}} \\ &\quad - 0.000118{\text{ Layer}}\;{\text{thickness}}*{\text{Scan}}\;{\text{speed}} - 1.083{\text{ Layer}}\;{\text{thickness}}*{\text{Hatch}}\;{\text{spacing}} \\ &\quad + \, 0.0284{\text{ Scan}}\;{\text{speed}}*{\text{Hatch}}\;{\text{spacing}} \\ \end{aligned}$$

$${\text{R-sq}} = 77.33\% ,{\text{R-sq}}\left( {{\text{adj}}} \right) = 50.88\%$$

On the other hand, the mathematical model for top roughness (R^2^ = 93.66% and Adj. R^2^ = 86.26%) and side roughness (R^2^ = 94.67% and Adj. R^2^ = 88.46%) are presented.

4 $$\begin{aligned} {\text{Ra - Top}} & = - 83.5 + 0.120{\text{ Laser}}\;{\text{power}} + 1.318{\text{ Layer}}\;{\text{thickness}} + 0.0793{\text{ Scan}}\;{\text{speed}} \\ &\quad+ 103.5{\text{ Hatch}}\;{\text{spacing}} - 0.000212{\text{ Laser}}\;{\text{power}}*{\text{Laser}}\;{\text{power}} \\ &\quad - 0.00780{\text{ Layer}}\;{\text{thickness}}*{\text{Layer}}\;{\text{thickness}} - 0.000024{\text{ Scan}}\;{\text{speed}}*{\text{Scan}}\;{\text{speed}} \\ & \quad- 178.3{\text{ Hatch}}\;{\text{spacing}}*{\text{Hatch spacing}} - 0.00061{\text{ Laser}}\;{\text{power}}*{\text{Layer}}\;{\text{thickness}} \\ &\quad - 0.000025{\text{ Laser power}}*{\text{Scan speed}} + 0.015{\text{ Laser}}\;{\text{power}}*{\text{Hatch}}\;{\text{spacing}} \\ & \quad- 0.000151{\text{ Layer}}\;{\text{thickness}}*{\text{Scan}}\;{\text{speed}} - 0.518{\text{ Layer}}\;{\text{thickness}}*{\text{Hatch}}\;{\text{spacing}} \\ & \quad- 0.0074{\text{ Scan speed}}*{\text{Hatch}}\;{\text{spacing}} \\ \end{aligned}$$

$${\text{R-sq}} = 93.66\% ,{\text{R-sq}}\left( {{\text{adj}}} \right) = 86.26\%$$

5 $$\begin{aligned} {\text{Ra - Side}} & = 93.5 - 0.077{\text{ Laser power}} + 0.33{\text{ Layer}}\;{\text{thickness}} - 0.0282{\text{ Scan speed}} \\ &\quad+ \, 49{\text{ Hatch}}\;{\text{spacing}} - 0.000392{\text{ Laser}}\;{\text{power}}*{\text{Laser}}\;{\text{power}} \\ & \quad- \, 0.0001{\text{ Layer}}\;{\text{thickness}}*{\text{Layer}}\;{\text{thickness }} + \, 0.000011{\text{ Scan speed}}*{\text{Scan speed}} \\ & \quad+ \, 55{\text{ Hatch}}\;{\text{spacing}}*{\text{Hatch}}\;{\text{spacing}} + 0.00176{\text{ Laser}}\;{\text{power}}*{\text{Layer thickness}} \\ & \quad+ \, 0.000055{\text{ Laser power}}*{\text{Scan speed}} - 0.112{\text{ Laser}}\;{\text{power}}*{\text{Hatch spacing}} \\ & \quad- \, 0.000388{\text{ Layer}}\;{\text{thickness}}*{\text{Scan speed}} - 0.02{\text{ Layer}}\;{\text{thickness}}*{\text{Hatch spacing}} \\ &\quad - \, 0.0133\;{\text{Scan speed}}*{\text{Hatch spacing}} \\ \end{aligned}$$

$${\text{R-sq}} = 94.67\% ,{\text{R-sq}}\left( {{\text{adj}}} \right) = 88.46\%$$

**Figure 3:**
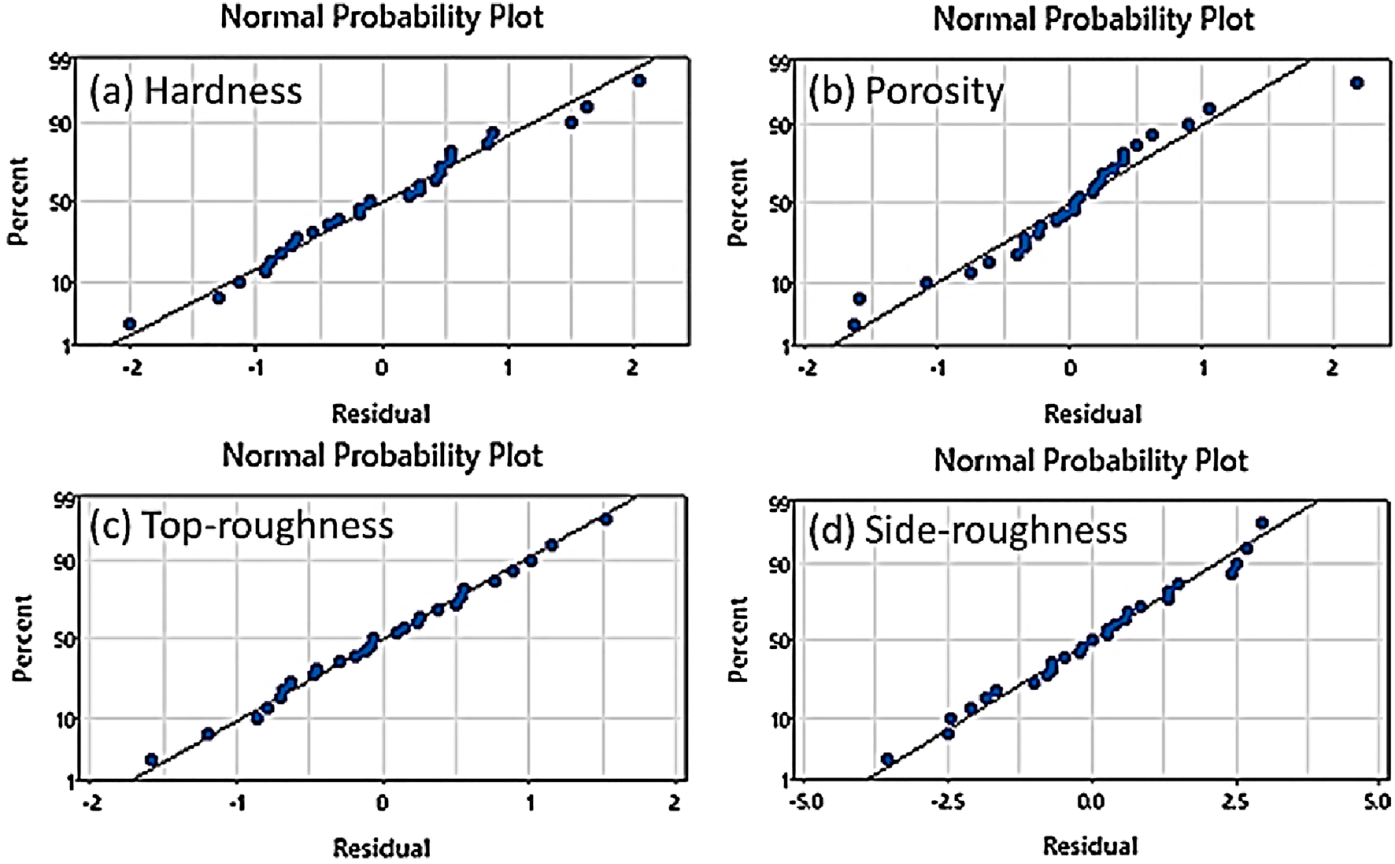
Residual plot of predictive models of L-PBF process showing (**a**) hardness (**b**) porosity (**c**) Top-roughness and (**d**) side-roughness.

For all responses, the statistical significance of the presented models is defined through *p*-value < 0.05 as described in parametric influence analysis. The proposed models are examined through comparison of predicted versus actual plots and normality residual plots. The normal probability plots of all models are shown in Fig. 3. The normal plot of hardness is presented in Fig. 3a where most of the data pints are on-line depicting the normal error distribution. Similarly, the data points for porosity (Fig. 3b) are distributed on-line representing good agreement of the percentile and residual data^32^. The model adequacy as presented before shows the statistical significance and reliability for predicting top roughness (Fig. 3c) and side-roughness (Fig. 3d). Conclusively, the normal distribution of the data points around the line presents the fitting of model and minimal error.

#### Multi objective optimization of process parameters by COPRAS approach

Multiple-objective optimization can be used to obtain mutually optimal parameter combinations. The major challenge in multi objective optimization is determining an accurate weight for the output response. Researchers determined the weight to be assigned to the response based on their experience and the trial & error method they used to determine the parameter's significance. In order to compute the weightage for the output response, a newer approach needs to be developed. A multi-criteria decision making problem was solved earlier weights for assigning individual weights to the output response. In many cases, decision makers express their ideas by taking into account choice variables in order to determine the weights for their traits and to make parallel comparisons with real-world situations. In the presence of equally conflicting factors, the COmplex PRoportional ASsessment (COPRAS) technique incorporates proportionate and direct confidence on the relevance and efficacy of accessible substitutes. COPRAS connects the weights by ranking and suggests the best ideal parameters by taking into account the effectiveness of alternatives in respect to many control aspects. These steps were illustrated as part of the COPRAS approach.

***Step 1:*** The decision matrix must first be created before the output parameter might be normalized (Table 4).

6 $$NOij=\frac{Qij}{\sum_{i=1}^{m}Qij}\; ij=1 \ldots n$$

where NOij is the normalized matrix, Qij is constructed matrix based on output response

**Table 4:** Normalized and weighted normalized matrix values.

Trail	Input Parameter				Normalized matrix				Weighted normalized matrix				Pi	Ri
	Laser Power (W)	Hatch spacing (mm)	Scanning Speed (mm/s)	Layer thickness (μm)	Hardness (HV)	Porosity (%)	Ra (μm)-Top	Ra (μm)-Side	Hardness (HV)	Porosity (%)	Ra (μm)-Top	Ra (μm)-Side		
1	250	0.1	1000	30	0.1128	0.0866	0.1049	0.1077	0.0282	0.0217	0.0262	0.0269	0.0282	0.0748
2	250	0.2	1300	40	0.1085	0.1484	0.1264	0.1172	0.0271	0.0371	0.0316	0.0293	0.0271	0.0980
3	250	0.3	1600	50	0.1064	0.1992	0.1468	0.1254	0.0266	0.0498	0.0367	0.0314	0.0266	0.1178
4	300	0.1	1300	50	0.1138	0.0405	0.0913	0.1063	0.0285	0.0101	0.0228	0.0266	0.0285	0.0595
5	300	0.2	1600	30	0.1096	0.1225	0.1209	0.1141	0.0274	0.0306	0.0302	0.0285	0.0274	0.0894
6	300	0.3	1000	40	0.1074	0.1679	0.1406	0.1189	0.0269	0.0420	0.0352	0.0297	0.0269	0.1069
7	350	0.1	1600	40	0.1149	0.0602	0.0800	0.1014	0.0287	0.0151	0.0200	0.0254	0.0287	0.0604
8	350	0.2	1000	50	0.1106	0.1059	0.1157	0.1124	0.0277	0.0265	0.0289	0.0281	0.0277	0.0835
9	350	0.3	1300	30	0.1160	0.0687	0.0733	0.0966	0.0290	0.0172	0.0183	0.0242	0.0290	0.0597

***Step 2:*** Equal weightage is given to all the output response. Calculated individual weight is multiplied to a normalized decision matrix in this process. Here, the normalized matrix (Eq. 6) will be multiplied by the weightage computed Equal Weight (We) to create the normalized weighted matrix (NWij) shown (We = 0.25) in Table 4.

7 $$NWij = We \times NOij$$

***Step 3:*** Pi calculation: Pi represents maximization function (Pi) calculated by Eq. (8).

8 $$Pi = \sum_{j=1}^{n}Qij$$

where n is total maximize response.

***Step 4:*** Ri calculation: Ri represent minimization function and n represent number of response, that are calculated using Eq. 13. Normalized and weighted normalized matrix in COPRAS approach are outlined in Table 4.

9 $$Ri = \sum_{j=m+1}^{n}Qij$$

***Step 5:*** Spotting the diminutive value (Rmin) of R

10 $${\text{Rmin}} = {\text{min\, Ri}}$$

***Step 6:*** Determination of relative weight of output values (Qi).

The Qi values are computed by using Eq. (11) and the highest value in Qi will be denoted as Qmax.

11 $${\mathrm{Qi}} = {\mathrm{Pi}}+\frac{\mathrm{Rmin }\sum_{\mathrm{J}=1}^{\mathrm{m}}{\mathrm{Ri}}}{{\mathrm{Ri}}\sum_{\mathrm{j}=1}^{\mathrm{m}}{\mathrm{Rmin}}/{\mathrm{Ri}}}.$$

***Step 7:*** Determination of the utility degree (Ni) × 

12 $${\mathrm{Ni}} = 100 \times ({\mathrm{Qi}}/{\mathrm{Qmax}})$$

Maximum value is rated as the ideal parameter based on utility degree, and the derived values are shown in Table 5.

**Table 5:** Ranking and Optimality criterion.

Trail	Ri_min_/Ri	Qi	Ni (%)	Ranking
1	0.7954	0.1156	83.3946	4
2	0.6073	0.0938	67.7117	7
3	0.5051	0.0821	59.2275	9
4	1.0000	0.1383	99.8034	2
5	0.6658	0.1005	72.5437	6
6	0.5569	0.0881	63.5303	8
7	0.9851	0.1370	98.8115	3
8	0.7129	0.1060	76.4691	5
9	0.9976	0.1386	100.0000	1

### Parametric effect analysis

**Figure 4:**
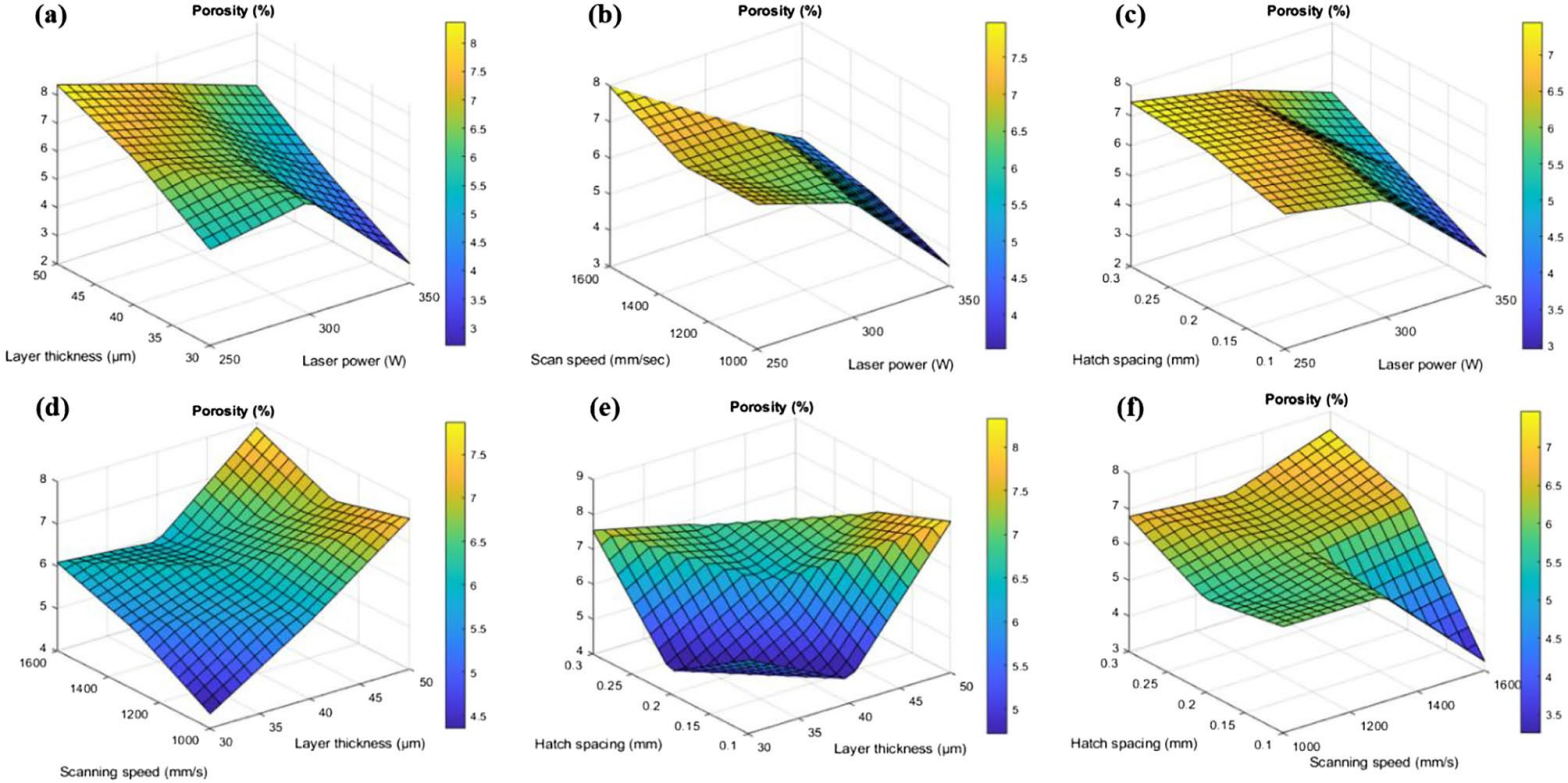
3D surface interaction plots for porosity analysis for (**a**) layer thickness and laser power (**b**) scan speed and laser power (**c**) hatch spacing and laser power (**d**) scanning speed and layer thickness (**e**) hatch spacing and layer thickness and (**f**) hatch spacing and scanning speed.

Figure 4 shows the 3D surface plots for porosity based on the interaction of the input parameters. Figure 4a shows the interaction among the layer thickness and laser power.

#### Porosity analysis

**Figure 5:**
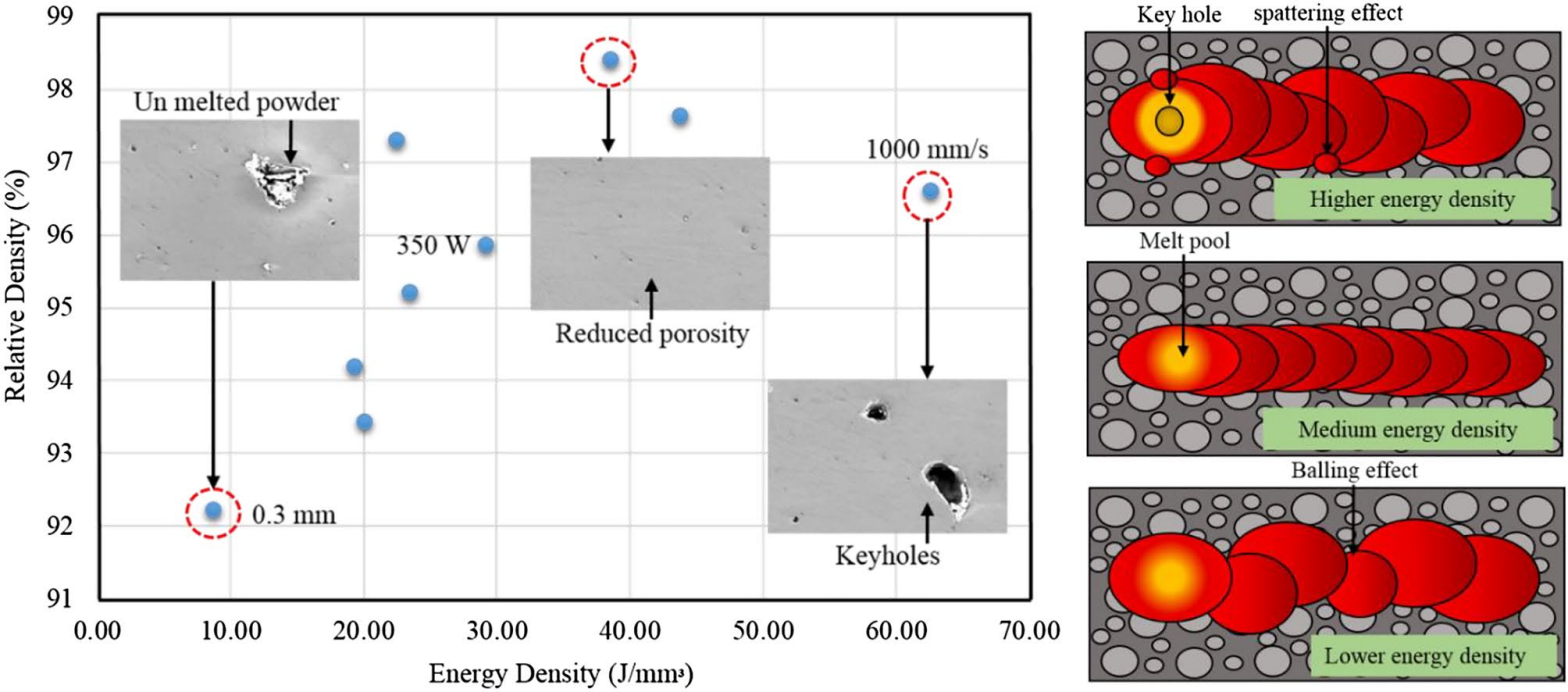
Interaction plots with schematic diagram for the relative density and energy density with respect to hatch spacing, scanning speed and laser power.

**Figure 6:**
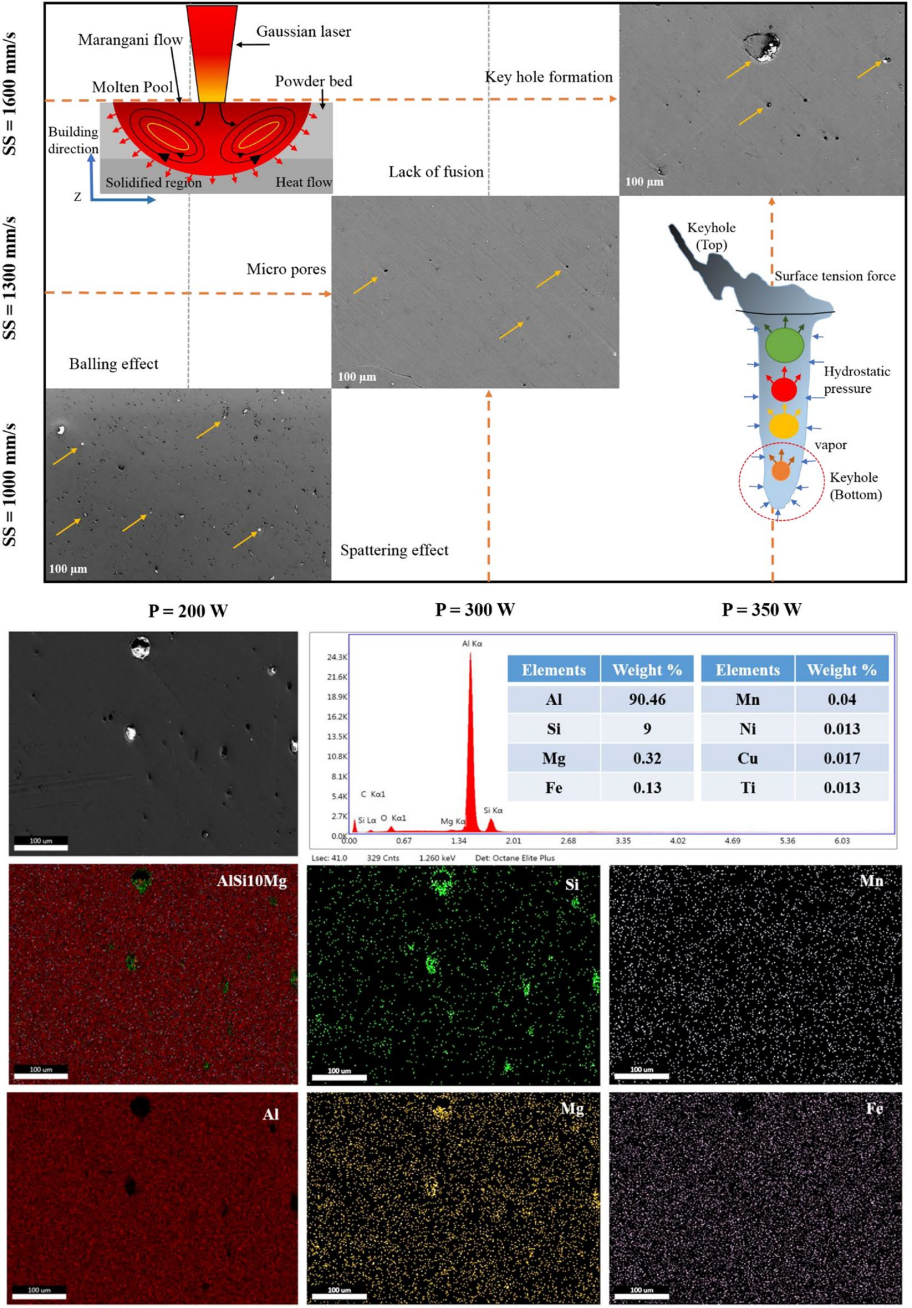
FE-SEM interaction plots with EDAX mapping for the laser power and scanning speed.

**Figure 7:**
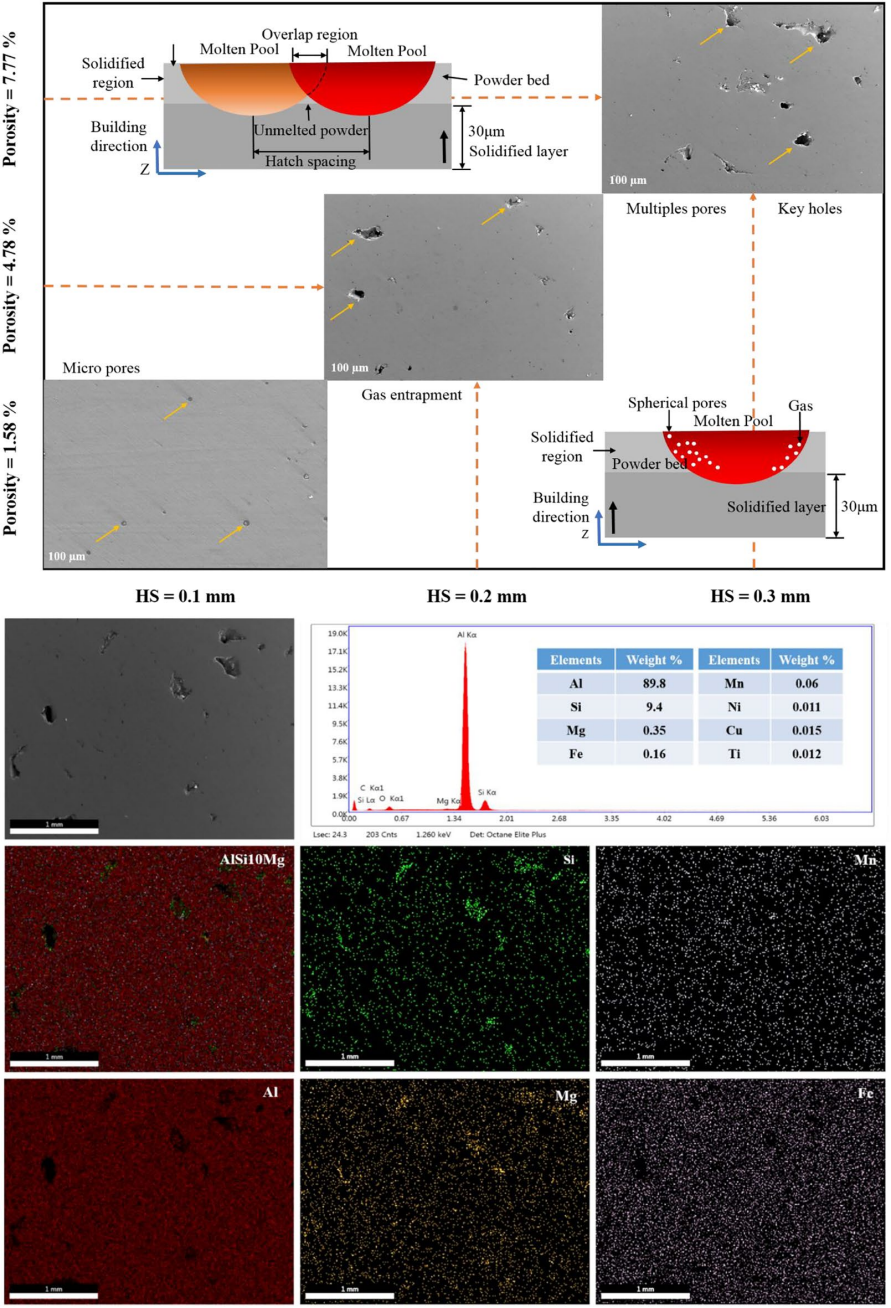
FE-SEM interaction plots with EDAX mapping for the hatch spacing and porosity.

It was observed that the porosity reduces with decrease in the layer thickness. This was due to the fact that the reduction in the layer thickness increases the overlapping region which leads to uniform distribution of thermal gradient (Fig. 5). Similarly, the porosity will be reduced by the re-melting of melt pools, especially in the lower hatch spacing (0.1 mm) as shown in the Fig. 7. It was also noted that the increase in laser power reduces the porosity of the printed parts, because the maximum laser power creates a sufficient energy for the fusion of particles and that will reduces the porosity which was shown in the Fig. 6. Similarly, lower laser power does not possess enough heat for the perfect fusion of the particles which leads to the occurrence of high porosity (Fig. 6). According to Weingarten et al.^33^, the water in the original powder is the source of the hydrogen in the pores, and the gas in the spherical pores is primarily hydrogen, this observation was shown in Fig. 5 as schematic diagram. This kind of entrapment of gas may induce the porosity formation. On the interaction plot it was noted that minimum porosity of 2 to 3% was observed on the minimum layer thickness of 30 µm and laser power of 350W. The increase in scanning speed increases the porosity of the printed parts, because increase in scanning speed does not provides sufficient time for the fusion of particles, improper fusion acts as the porous region and that affects the structural integrity which was shown in Fig. 5. Similarly decrease in scanning speed also increases the porosity, as the prolonged exposure of laser may leads to the keyhole formation or spattering effect (Fig. 5). Khorasani et al. stated that reduced hardness and density that are related to wettability and Rayleigh instability were attained during the higher scan speed and also specified that using the Eq. (13), the melt pool temperature can be calculated^34^.

13 $${T}_{mp}= \frac{{C}_{2}}{\lambda {\ln}\left(\frac{{C}_{1}{S}_{S}{H}_{S}}{{L}_{p}{\delta }^{5}}+1\right)}$$

*S*_*S*_, *H*_*S*_, and *L*_*p*_ represents the scanning speed, hatch spacing and laser power, λ is laser wavelength of the laser, ${T}_{mp}$ is the temperature of the melt pool and C1, C2 represented as Planck distribution.

On the interaction plot of Fig. 4b, minimum porosity of 3 to 4% was observed at maximum laser power of 350W and at the optimum scanning speed of 1100 to 1300 mm/s. Similar to this, Qiu et al. discovered that speeding up the scanning creates a more stable melting pool and a continuous, homogeneous material and that enhances the strength^35^. The increase in hatch spacing increases the porosity because distance between the two adjacent melt pools increases causing the reduction in the overlapping region of the melt pools and this was explained in the schematic diagram in the Fig. 7. This will leads to the improper fusion of particles which results in balling effect^36^, the schematic diagram of balling effect was shown in Fig. 5. This phenomenon shows good agreement with the interaction plots from Fig. 4c, which shows reduced porosity of 2 to 3% with the minimum hatch spacing distance of 0.1 mm and with the maximum laser power of 350 W. Interaction plot from Fig. 4d shows minimum porosity of 4% with the minimum layer thickness of 30 µm and with the scanning speed of 1000 mm/s. The accumulation of significant heat increases the likelihood of the balling phenomenon or other defects, which have a negative impact on the morphologies near the edges of the parts. As a result, lowering laser energy density helps to reduce heat accumulation, which reduces the negative effect on edge morphology^37^. The results from Yang et al. show a clear difference in molten pool morphology when the energy density is changed. As the energy density increases, so does the depth and width of the molten pool. Furthermore, the increase in molten pool depth is more significant when compared to the width of the molten pool^38^. Based on the thermocapillary effect, higher scan speed attributes to the lower temperature in the melt pool which consequently, the value of surface tension for the solid–liquid phase and the liquid–gas phase will become higher and that can be obtained from the Eq. 14. This phenomenon proves that the melt pool temperature and the scan speed is inversely proportional and according to Eq. (15), there is a likelihood of proliferation in the formation of droplets^34^.

14 $$\gamma = {\gamma }^{*}\left(\frac{{T}_{C}-{T}_{0}}{{T}_{C}}\right){\left[1-\left[\frac{{C}_{2}}{{T}_{C}-{T}_{0}\lambda {\ln}\left(\frac{{C}_{1}{S}_{S}{H}_{S}}{{L}_{p}{\delta }^{5}}+1\right)}- \frac{{T}_{C}}{{T}_{C}-{T}_{0}}\right]\right]}^{n}$$

15 $$S= {\gamma }_{SG}-\left({\gamma }_{SL}+{\gamma }_{LG} \right),\quad {\mathrm{If\; s}} > 0 = {\mathrm{Film\, appears}}. \,{\mathrm{If s}} < 0 = {\mathrm{Droplet appears}}$$

T_0_ and T_C_ represents the reference value and critical value for the temperature, for each liquid  ${\gamma }^{*}$ is constant^34^.

This confirms that the reduced layer thickness has the significant role in reducing the porosity of the printed parts. Similarly, the interaction among the hatch spacing and layer thickness from the Fig. 4e represents that the minimum porosity of 4% was observed during the 0.2 mm hatch spacing and 40 µm layer thickness and finally on the interaction of hatch spacing and scanning speed from the Fig. 4f shows reduced porosity of 3% with the hatch spacing of 0.1 mm and scanning speed of 1600 mm/s. This approves that the minimum hatch spacing has the predominant influence over the reduction of porosity which was shown in Fig. 7.

#### Hardness analysis on the SS

**Figure 8:**
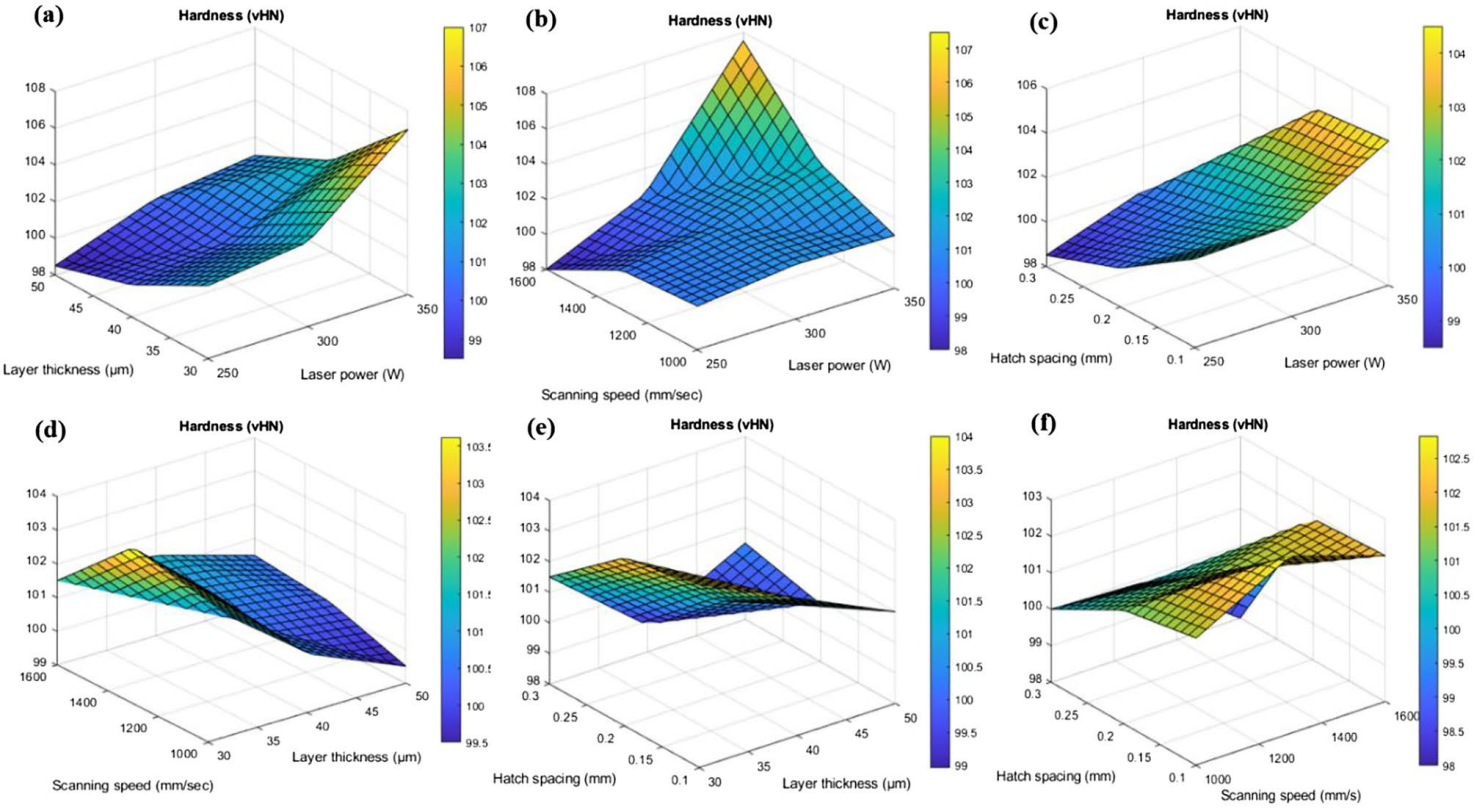
3D surface interaction plots for hardness analysis for (**a**) layer thickness and laser power (**b**) scanning speed and laser power (**c**) hatch spacing and laser power (**d**) scanning speed and layer thickness (**e**) hatch spacing and layer thickness and (**f**) hatch spacing and scanning speed.

Figure 8 shows the 3D surface plots of micro hardness based on the interaction of the input parameters.

Laser power was one of the significant parameter which affects the micro hardness of the printed parts. Similarly, layer thickness also possess some key feature such as proper fusion of particles, overlapping of melt pools, even growth of precipitates which may influence the hardness of the printed parts^39^. The activity of adhesion is a significant aspect in the rheology of the melting pool that influences the porosity and hardness. Equation 16 is obtained in solid–liquid interfaces such as the interaction between the melting pools and the solidified layers using Young's law and Young-Dupre’s relation^34^.

16 $${W}_{\alpha }= \gamma (1+\cos\theta )$$

where the meniscus's radius is determined by its distance from the surface's normal line and it is denoted by $\theta$. This relation elucidates the interaction between the work of adhesion and the surface tension. It states that the reduction in the adhesion effect was mainly due to the lower hatching spaces. This characteristics was also leads to the decrease in the surface tension between the melt pool and the solidified layer, which was based on the Planck’s distribution and thermocapillary effect. Further, there will an unstable melt pool with positive integer of fluid flow gradient and that was due to the high intensity of laser beam (Eq. 17)^40^.

17 $$\nabla \bar{u}_{fb}=\frac{\partial {u}_{fb}}{\partial x}\hat{\mathrm{i}}+\frac{\partial {u}_{fb}}{\partial y}\hat{\mathrm{j}}+\frac{\partial {u}_{fb}}{\partial y} \hat{k} \ne 0$$

This characteristic causes the generation of waves which entraps the unmelted powders and that will leads to the formation of porosities in the subsequent layers.

From 3D surface plot of Fig. 8a, it was noted that the hardness of the printed parts increases with increase in the laser power. Micro hardness values varies from 108 to 109 VHN which is the maximum hardness obtained for the laser power of 330 to 350W. Similarly, decrease in layer thickness increases the micro hardness of the printed parts. This was due to the over lapping of melt pools which causes the re-melting and influences the grain refinements and finally it attributes to the enhanced hardness^41^. The fluidity of the molten liquid and the depth of the molten pool have the most effects on the sample density during the selective laser melting process. The viscosity of the molten liquid decreases as the temperature of the molten pool rises, improving fluidity. The bonding efficiency between the present layer and the created layer is dependent on the depth of the molten pool^42^. Maximum hardness of 103 to 105 VHN was observed over the layer thickness of 35 to 30 µm. On the interaction of scanning speed and laser power in the Fig. 8b, it was found that scanning speed around 1400 to 1200 mm/s has the considerable effect in increasing the hardness value of 101 to 103 VHN. Increase in scanning speed reduces the exposure time of the fusion process which attributes to improper fusion of particles (Fig. 7). This characteristic increases porosity and decreases density and hardness that are considered to be prevalent defects in metal AM^34^. It also causes low wettability and an increased probability of balling effect which was represented by schematic diagram in the Fig. 5. Similarly, lower scanning speed leads to the prolonged exposure to the laser beam and that makes multiple defects like spattering effect, keyhole formation. However, the maximum hardness value of 108 to 109 VHN was noted on the interaction of 1600 mm/s scanning speed and 350W laser power. Figure 8c shows the interaction of hatch spacing and laser power. It was found that the decrease in the hatch spacing increases the micro hardness of the printed parts. This was due to the fact that the overlapping of melt pools and re-melting leads to the grain refinements due to the homogenous distribution of thermal gradient and thereby reducing the residual stress. The marangoni flow has an important role in the melt pool formation. The schematic representation of marangoni flow was shown in the Fig. 6. Khorasani et al. stated that the Eqs. 18 and 19 helps to achieve a stable melt pool with minimum porosity and maximum hardness.

18 $${\mathrm{E}}_{\rm SL}={\gamma }_{SL}{S}_{c}$$

19 $${\mathrm{E}}_{\rm t} = {\mathrm{E}}_{1}+{\mathrm{E}}_{2} = {(\gamma }_{1} - {\gamma }_{2}){S}_{c}$$

E_1_ and E_2_ is energy of the melt pool and the solidified layer and S_c_ represents the solidified melt pool’s contact area^34^. Hatch spacing of 0.10 to 0.15 mm shows the maximum hardness value of 95 to 93 VHN. But on the interaction of 340W laser power and 0.10 mm hatch spacing, the maximum hardness value of 106 VHN was obtained. Figure 8d represents the interaction of scanning speed and layer thickness. It was found that the maximum hardness of 104 VHN was observed on the scanning speed of around 1250 mm/s to 1450 mm/s and the layer thickness of 30 to 35 µm. This interaction proves that the lower layer thickness with optimum scanning speed enhances the hardness of the printed parts. On the interaction plot Fig. 8e, it was found that the maximum hardness value of 104 VHN was observed in the minimum hatch spacing of 0.1 mm and minimum layer thickness of 30 µm. This interaction confirms that the improvement in the hardness will be attained only by reducing the layer thickness and hatch spacing. Khorasani et al. also found that the pressure in the droplet can be examined using the Eq. (20), where R_drop_ is the droplet radius^40^.

20 $${\mathrm{P}}_{1} = {\mathrm{P}}_{0}-{\frac{2}{{R}_{drop}}\gamma }^{*}\left(\frac{{T}_{C}-{T}_{0}}{{T}_{C}}\right){\left[1-\left[\frac{{C}_{2}}{{T}_{C}-{T}_{0}\lambda \mathrm{ln}\left(\frac{{C}_{1}{S}_{S}{H}_{S}}{{L}_{p}{\delta }^{5}}+1\right)}- \frac{{T}_{C}}{{T}_{C}-{T}_{0}}\right]\right]}^{n}$$

**Figure 9:**
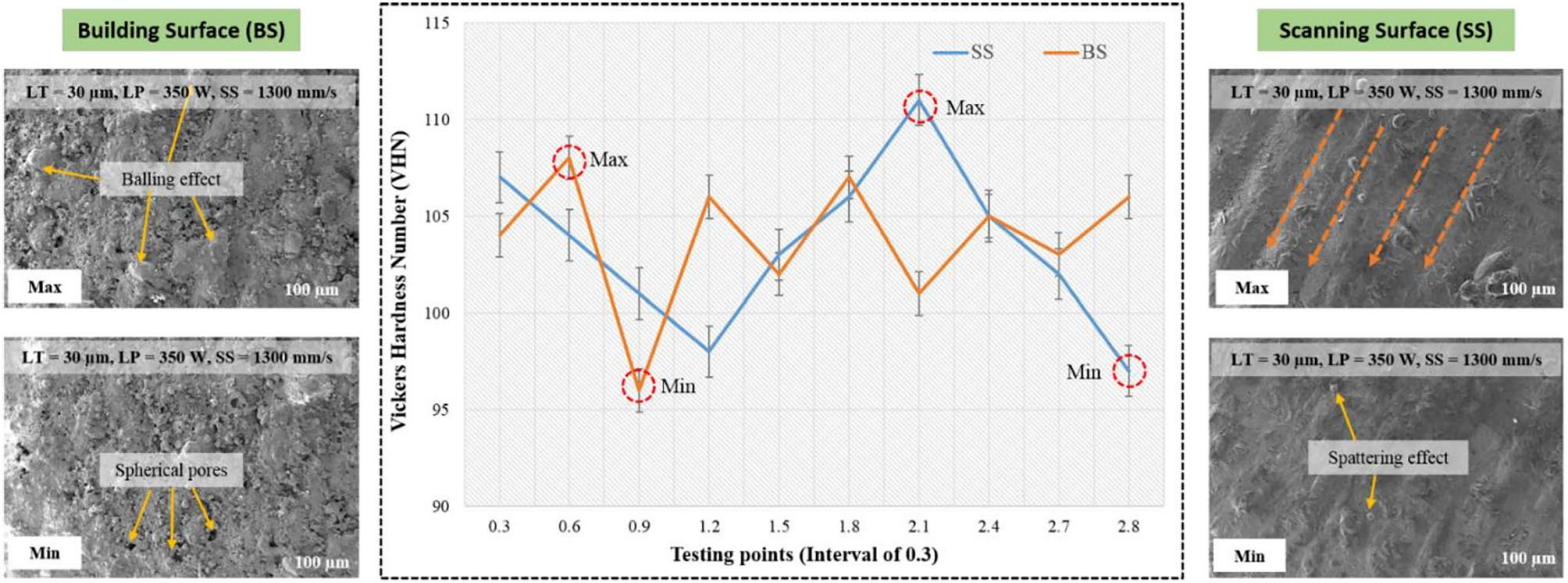
Hardness plots along the building surface (BS) and scanning surface (SS) at the interval of 0.3 mm.

According to Eq. (8), the droplet’s pressure increases with lower scan speed and hatch spacing, which in turn raises the surface tension owing to thermo capillary effects and increases the possibility of bursting and dross. This increases the likelihood of porosity for succeeding layers. Figure 8f, shows the interaction of hatch spacing and scanning speed, it was observed that the maximum hardness of 104 to 105 VHN was evident in the minimum hatch spacing of 0.1 mm and optimum scanning speed ranges from 1200 to 1400 mm/s. This interaction of scanning speed and hatch spacing shows good agreement and satisfies the previous constrains of inputs parameters. Figure 9 shows the hardness plots along the building surface and scanning surface at the interval of 0.3 mm. The ups and downs in the hardness plots justifies the hatch spacing in the scanning surface (SS) and layer thickness in the building surface (BS).

#### Roughness analysis

**Figure 10:**
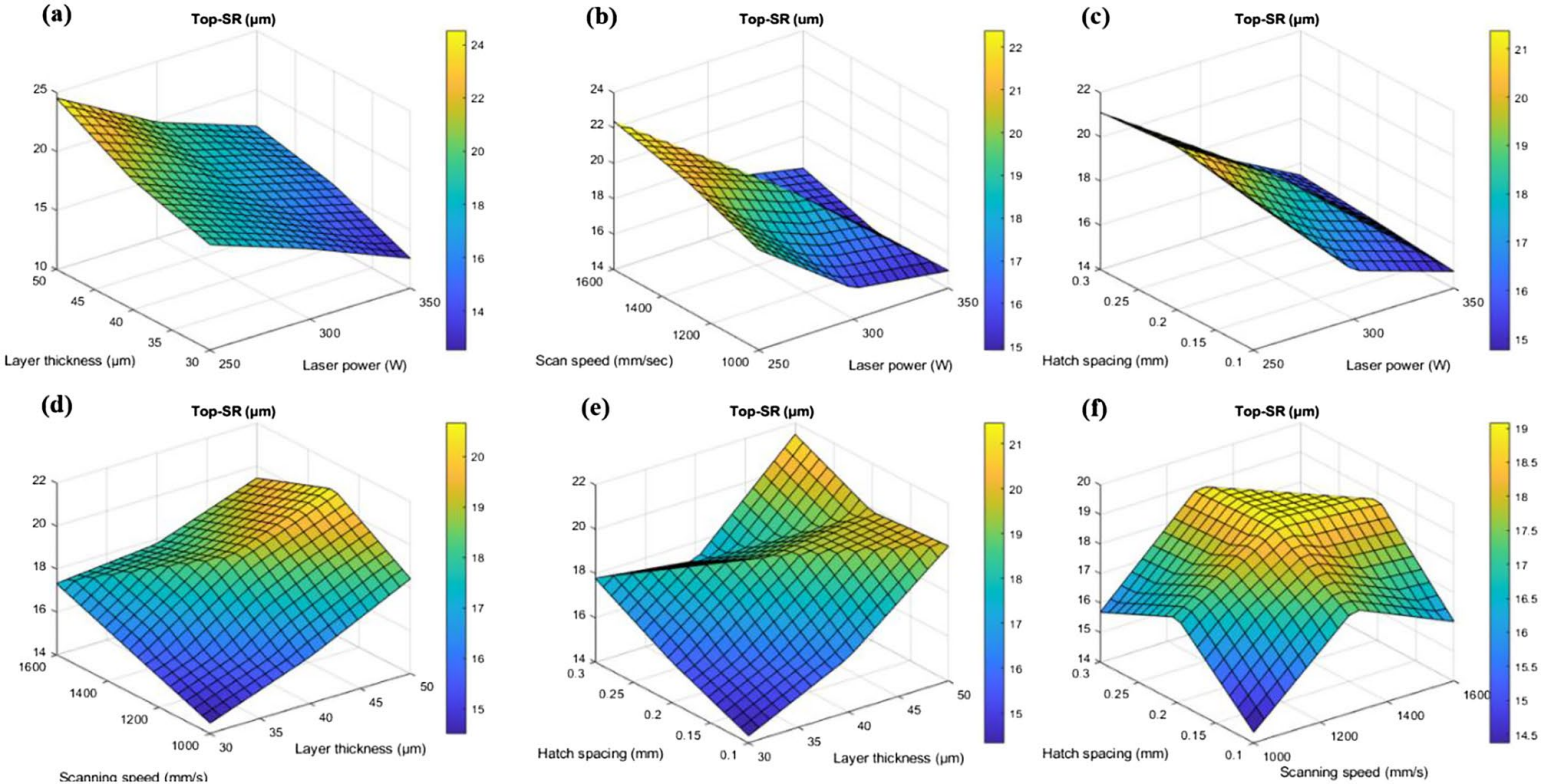
3D surface interaction plots for (**a**) layer thickness and laser power (**b**) scanning speed and laser power (**c**) hatch spacing and laser power (**d**) scanning speed and layer thickness (**e**) hatch spacing and layer thickness and (**f**) hatch spacing and scanning speed.

**Figure 11:**
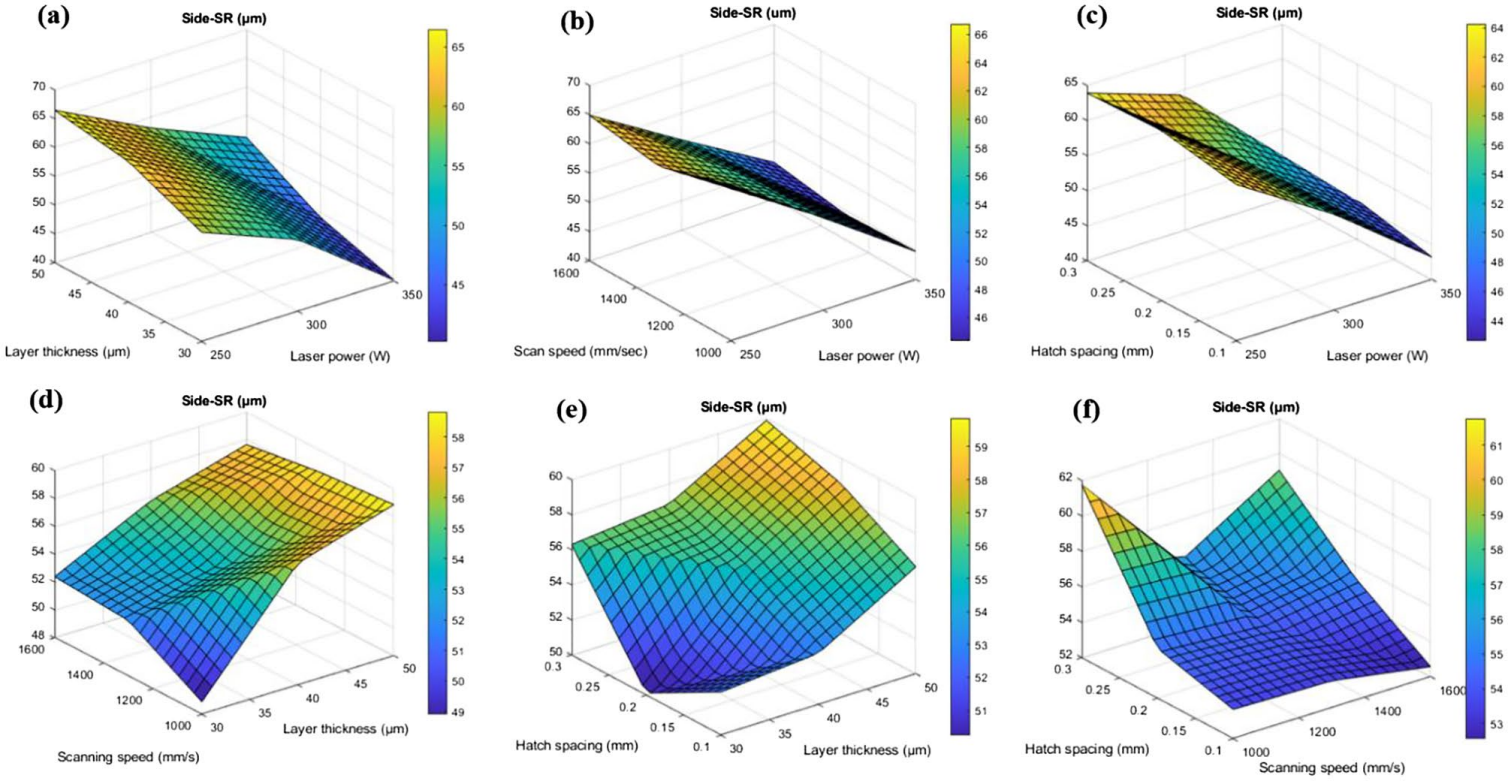
3D surface interaction plots for (**a**) layer thickness and laser power (**b**) scanning speed and laser power (**c**) hatch spacing and laser power (**d**) scanning speed and layer thickness (**e**) hatch spacing and layer thickness and (**f**) hatch spacing and scanning speed.

Figures 10a-f and 11a-f show the surface plots of top and side surface roughness based on the interaction of input parameters.

**Figure 12:**
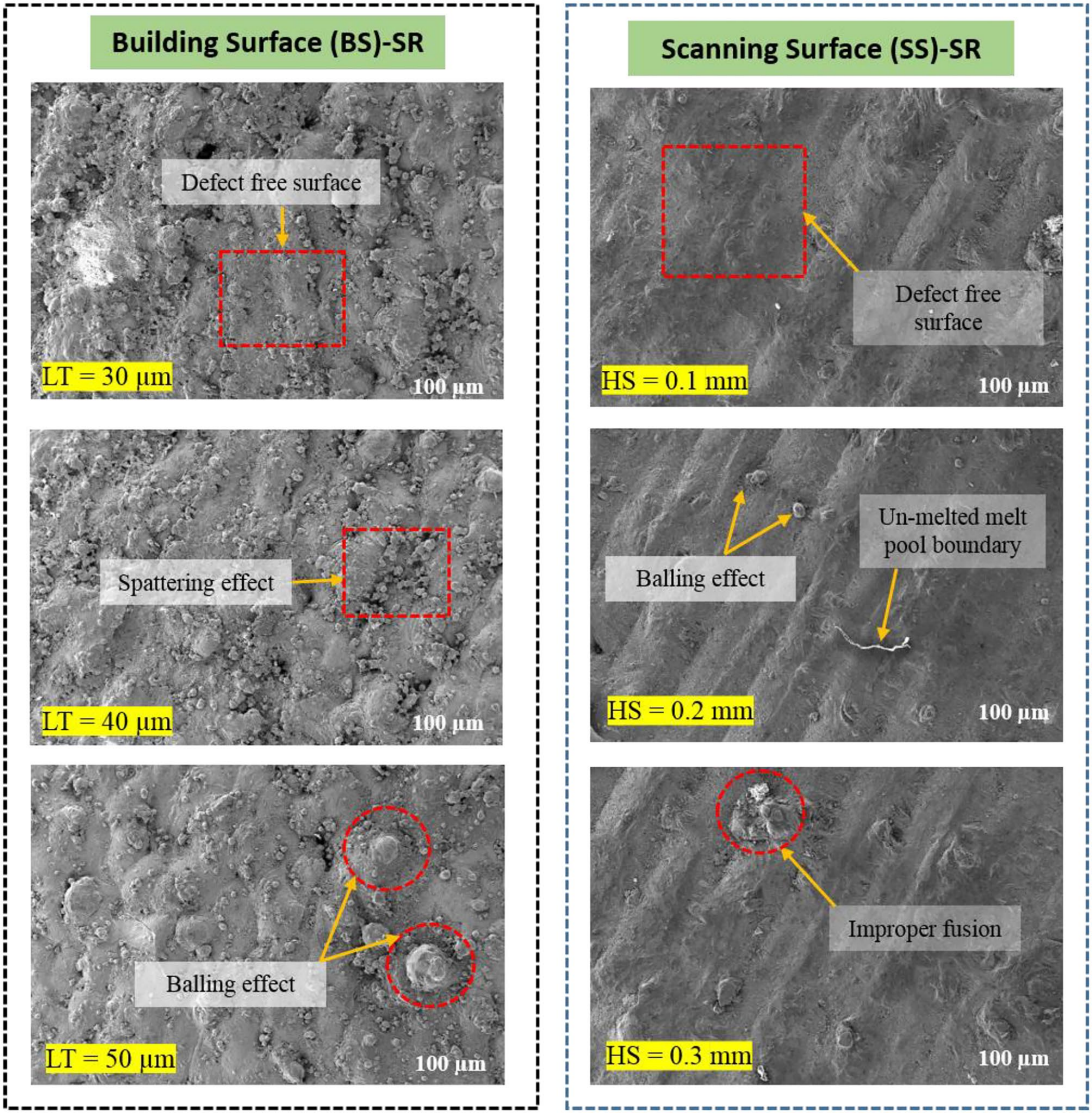
Surface defects comparison between the building and scanning surface with respect to layer thickness and hatch spacing.

Figures 10a and 11a show the interaction among the layer thickness and laser power on the top and side surface roughness. On the basis of interaction among the layer thickness and laser power, increase in layer thickness increases the surface roughness of the top surface as well as the side surface. This was due to the fact that the improper fusion of particles may takes place due to the increased layer thickness and this can be observed from the un-melted powders on the printed parts^43^. The liquid doesn't entirely spread as it solidifies due to the overly fast cooling rate, which leads to flaws like asymmetrical pores^44^. In case of laser power, reduced laser power (< 300W), leads to the improper melt pool formation which consequently attributes to the increase in surface roughness in both top and side surface. The minimum top surface roughness of around 11 µm and side surface roughness of about 40 µm was observed on the reduced layer thickness of 40 µm and maximum laser power of 350W and this was substantiated in the Fig. 12. However, if the laser power reaches more than 350W there will be high possibility of keyhole formation and that affects the surface characteristics of the printed parts which was reported in the Fig. 6 with the schematic representation of keyhole formation. Khorasani et al. emphasized**,** the formation of Marangoni’s effects during the melt pool formation, in which it transfers the heat to the adjacent regions of the melt pool. As a result, a melt pool region (mushy zone) of partially solid and liquid material develops. The gradient for the melt flow is zero in the mushy state which attributes to the formation of thermal stress due to the restoration of higher temperature at that region and also led to the reduction in the surface tension. Equation 21 represents that increase in temperature increases the K value^45^.

21 $$k= \frac{T-{T}_{0}}{{T}_{c}- {T}_{0}}$$

Surface tension of the melt pools at its critical temperature can be evaluated by the Eq. 22 and also it was well known that except during surface vaporization, the surface tension will never reach zero.

22 $$\gamma = \sqrt[3]{\frac{{1}^{2}}{{v}_{1}}}(T-{T}_{c})$$

Due to the thermo-capillary effect, increasing in k in Eq. (21) causes the surface tension to decrease; this represents the Marangoni's effect and is illustrated in Eq. (23). Where γ ∗ is constant for each liquid^34^.

23 $$\gamma = {\gamma }^{*}\left(\frac{{T}_{C}-{T}_{0}}{{T}_{C}}\right){\left[1-k\right]}^{n}$$

**Figure 13:**
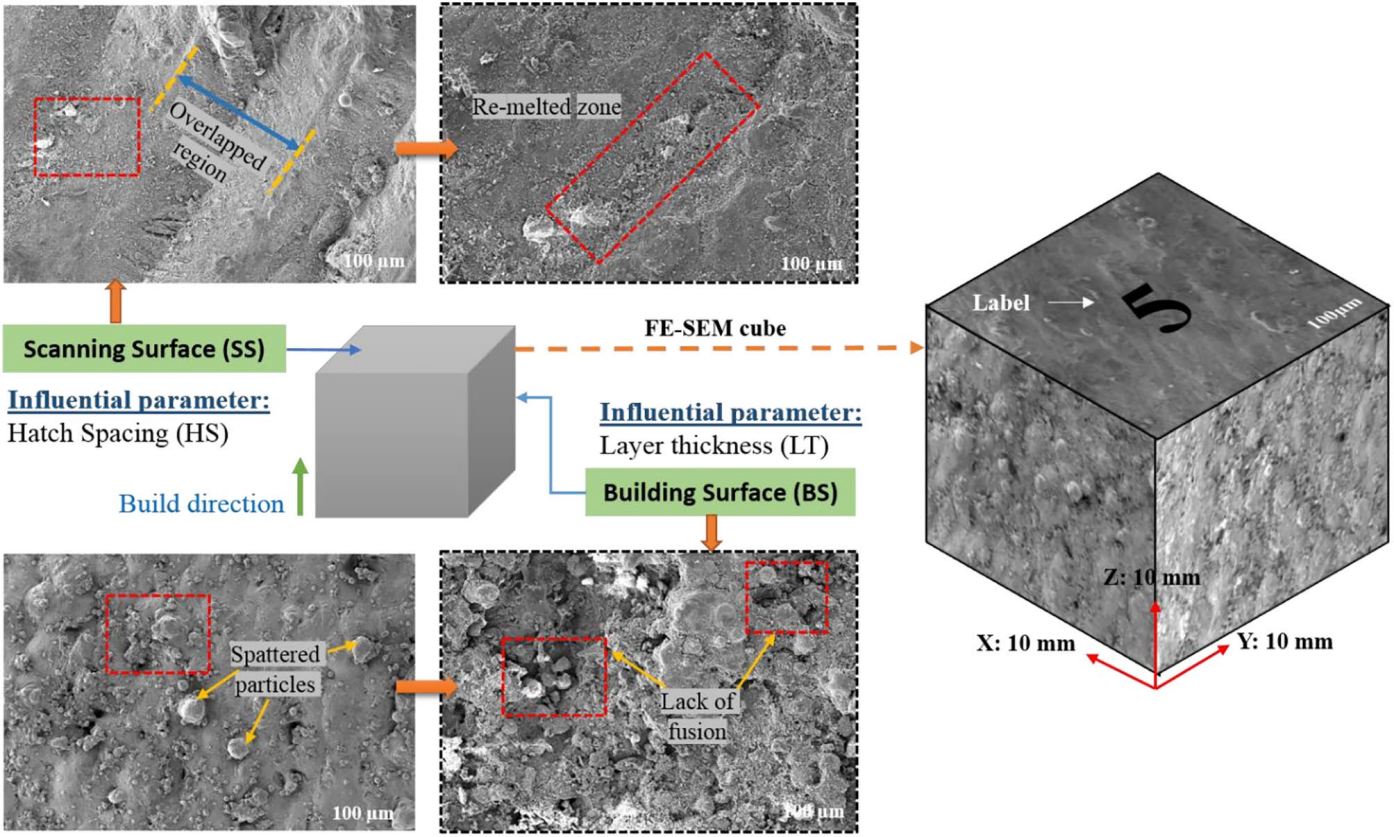
Comparison of scanning and building surface FE-SEM images of as-build AlSi10Mg parts based on the influential parameters.

The interaction between the vapour pressure and the surface tension due to hydrostatic force was more unstable due to the increase in the intensity of the laser beam. This was one of the important criteria for the formation of key holes which affects the mechanical properties. This phenomenon was represented schematically in Fig. 6. The scanning speed was also has an influential effect over the top and sider surface roughness of the printed parts. The increase in scanning speed decreases the exposure time of layer and that will creates a poor melt pool formation and an improper binding of particles, these defects may cause high surface roughness and leads to poor surface finish. This characteristic was reported in the Fig. 12. Similarly, minimum scanning speed cause prolonged exposure of laser beam to the particular surface which in turns creates more porosity due to the vaporization of melt pools^46^. This was one of the major reason for losing the dimensional accuracy of the printed parts. In the study by Masiagutova et al. geometrical positioning of the various weld tracks is also an important issue that must be addressed in order to reduce surface roughness. According to the study's findings, the average roughness can be lowered from 40 to 10 µm^47^. Before solidifying, the liquid totally permeates the molten pool and the deeper molten pool creates a strong bonds between the layers and that was strong enough to significantly minimize porosity^48^. On the interaction plot of scan speed and laser power from Figs. 10b and 11b show that the minimum top surface roughness of about 14.5 µm and minimum side surface roughness of about 45 µm was evident on the optimal scanning speed of 1200 mm/s to 1300 mm/s and with the maximum laser power of 350W. The roughness variation was caused by the scan direction, gas flow direction, and wiper movement direction, according to Bao-Qiang et al. scanning strategies with rotation increments of 90 effectively reduced variation. The findings supported direct part orientation and placement and can help users minimize roughness further by optimising processes or simplifying post-processing procedures^49^. The hatch spacing has the considerable effect over the top surface roughness of the printed parts. Increase in hatch spacing increases the top surface roughness which was shown in the Fig. 12a-f. The overlapping of melt pools enhances the surface properties by elimination porosity, residual stress and attaining the grain refinements. So, minimum hatch spacing distance would be preferred for printing the parts with reduced surface roughness. On the interaction plot from Figs. 10c and 11c, it was confirmed that the reduced hatch spacing of 0.1 mm displays minimum top surface roughness of about 14.5 µm and side surface roughness of about 42.5 µm with the maximum laser power of 350W. The melt pool flow properties can be examined with the help of Reynolds number using the Eq. 24^50^.

24 $${\mathrm{R}}_{\rm e}=\frac{\rho {u}_{m}\omega }{\mu }$$

where μ represents the viscosity of the melt pool, u_m_ represents the scan speed, ω represents the melting pool width and ρ represents the density. The decreased width of the melt pool was due to the rise in the scanning speed which possesses higher cooling rate with the reduction in the melt pool viscosity. The characteristics reduces the turbulence flow by dropping the Reynolds number. This reduction in the turbulence avoids some common defects such as spattering effect and void formation^50^. On the interaction plot from the Fig. 8d and 9d, it was noted that minimum top surface roughness of about 14 µm and side surface roughness of about 49 µm was evident on the minimum scanning speed and layer thickness of about 1000 mm/s and 30 µm thickness. From this interaction it was confirmed that the surface roughness can be controlled by reducing both the scanning speed and layer thickness. From the Figs. 10e and 11e, it was observed that the minimum top surface roughness of 14 µm and side surface roughness 50 µm can be obtained on the reduced hatch spacing in the range of 0.2 mm to 0.1 mm and with the reduced layer thickness of 30 µm. This observation shows good agreement with the previous interaction plots on hatch spacing and layer thickness. On the interaction of hatch spacing and scanning speed in the Fig. 10f and 11f, it was noted that the minimum top surface roughness of about 14 µm can be obtained by adopting the reduced hatch spacing of 0.1 mm and minimum scanning speed of 1000 mm/s. Similarly, the minimum side surface roughness of out 52.5 µm can be achieved by the reduced hatch spacing of 0.1 mm and maximum scanning speed of 1600 mm/s. This demonstrates that the decrease in top surface roughness is mostly driven by the minimum hatch spacing and decrease in side surface roughness is mostly driven by the minimum layer thickness. Top surface roughness generally influenced by laser power, scanning speed and hatch spacing but side surface roughness mostly influenced by layer thickness which was influenced by the re-coater and it was demonstrated by the Fig. 13.

## Conclusions

The LPBF process parameters were examined and enhanced based on the surface characteristics of the LPBFed AlSi10Mg parts. A fitted line plot was used to validate the BBD mathematical model, which was designed to forecast the surface attributes of LPBFed AlSi10Mg components. The following conclusions were drawn from the investigation.

- The balling effect was considerably reduced by adopting minimum hatch spacing of 0.1 mm which creates better thermodynamic stability and that aids in attaining the perfect fusion of the particles and also avoids the occurrence of un-melted particles. Another interesting observation made during the analysis was that the hatch spacing of 0.1 mm increases overlapping regions of melt pools, thereby eliminating the balling effect.
- On evaluating the interaction between the scan speed and laser power, the achieved optimal energy density of 38.46 J/mm^3^ not only reduced the porosity percentage (< 1%) but also significantly reduced the spattering effects. This reduced energy density helps to reduce heat accumulation, which reduces the negative effect on edge morphology. Moreover, expanding the overlap between melt pool regions causes re-melting, which promotes grain refinement and, in turn, increases hardness up to 107 VHN, improving the structural integrity of printed parts.
- Avoiding keyhole formation was one of the important constrain to get an effective melt pool formation and that was accomplished by creating a stable thermal capillary convection with optimal laser power with sufficient scan speed. The laser power of 350W and scan speed of 1130 mm/s obtained from the composite desirability-based mathematical optimization helps in reduction of keyhole formations.
- SS surface roughness generally depends on laser power, scanning speed and hatch spacing but BS surface roughness mostly depends on layer thickness which was influenced by the re-coater. The laser power of 350 W, layer thickness of 30 µm, scan speed of 1133 mm/s, and hatch spacing of 0.1 mm helps to reduce the spattering and balling effects and also avoids keyhole formation in which these improvements contribute to attain the minimum SS roughness of 9.8 µm, minimum BS roughness of 38 µm.

## Data Availability

The datasets used and analyzed during the current study available from the corresponding author on reasonable request.
